# Late-onset secondary pigmentary glaucoma following foldable intraocular lenses implantation in the ciliary sulcus: a long-term follow-up study

**DOI:** 10.1186/1471-2415-13-22

**Published:** 2013-06-07

**Authors:** Shirley HL Chang, Wei-Chi Wu, Shiu-Chen Wu

**Affiliations:** 1 Department of Ophthalmology, Chang Gung Memorial Hospital, Chang Gung University, No. 5, Fu Shin St., Guei-Shan Hsiang, Taoyuan 333, Taiwan; 2 College of Medicine, Chang Gung University, Taoyuan, Taiwan

**Keywords:** Ciliary sulcus, Foldable intraocular lens, Pigmentary glaucoma

## Abstract

**Background:**

To review the long-term outcomes of eyes with secondary pigmentary glaucoma associated with the implantation of foldable intraocular lenses (IOL) in the ciliary sulcus.

**Methods:**

The study retrospectively reviewed a series of cases who developed secondary pigmentary glaucoma after cataract operations. Data were collected from cases that were referred between 2002 and 2011.

**Results:**

Ten eyes of 10 patients who developed secondary pigmentary glaucoma after foldable IOLs implantation in the sulcus were included in this study.

Intraocular pressure (IOP) elevation was present in 2 eyes (20%) within the first 2 weeks following the initial cataract operation. The onset of glaucoma was delayed in the other 8 eyes (80%); the average onset time in these eyes was 21.9 ± 17.1 months after the initial cataract operation. Six eyes (60%) received surgical treatment because of large fluctuations and poor control of IOPs. Only 3 eyes (30%) achieved final visual acuities better than 20/40.

**Conclusion:**

Secondary pigmentary glaucoma accompanying the implantation of a foldable IOL in the ciliary sulcus may present as acute IOP elevation during the early postoperative period or, more commonly, late onset of IOP elevation accompanied by advanced glaucomatous optic nerve damage. Despite treatment, the visual prognosis for these patients can be poor. Placing a foldable IOL in the ciliary sulcus could pose a threat to the vision of the patients and long-term follow-up of IOP in these patients is necessary.

## Background

Secondary pigmentary glaucoma has been reported in patients who have undergone posterior chamber intraocular lens (IOL) implantation during cataract surgery, especially when the IOL was implanted in the ciliary sulcus following rupture of the posterior capsule [1-12]. Secondary pigmentary glaucoma can be identified via pigment deposit on the corneal endothelium and IOL,and by heavy pigmentation of the trabecular meshwork. Mechanical rubbing between the foldable IOL and the posterior region of the iris following the placement of the IOL in the sulcus can contribute to the release of pigments [1-12]. This release may present as either pigment dispersion syndrome accompanied by normal intraocular pressure (IOP) or pigmentary glaucoma accompanied by IOP elevation, glaucomatous optic nerve neuropathy and visual field loss. Because the implantation of a foldable IOL has become a popular choice for lens implantation after phacoemulsification, a significant number of foldable IOLs may have been either inadvertently or intentionally placed in the ciliary sulci of cataract patients following the rupture of the posterior capsule.

Most reports regarding the development of secondary pigmentary glaucoma following the implantation of a foldable IOL in the sulcus are either case reports or small case series [3-5,7,10-12]. Only two studies involve a larger number of cases, one of 20 and the other of 30 subjects [8,9]. However, the follow-up periods over which the patients in these studies were monitored are generally short, and most of the patients involved in the aforementioned prior studies are Caucasian. To address these issues, the present study investigates the pattern of IOP elevation and secondary pigmentary glaucoma following the implantation of foldable IOLs in the ciliary sulci of a group of Chinese patients with at least 20 months of follow-up. Both acute-onset and late-onset pigmentary glaucoma following the placement of an IOL in the ciliary sulcus are examined.

## Methods

The present study was designed as a retrospective study. The study reviewed a series of consecutive cases that were referred to the glaucoma clinics at the Department of Ophthalmology at Chang Gung Memorial Hospital in Taoyuan, Taiwan following the development of secondary IOP elevation after the patients underwent cataract operations. Data were collected from cases that were referred between 2002 and 2011. The Chang Gung Medical Foundation Institution Review Board approved the study.

Cases with secondary IOP elevation following phacoemulsification and foldable IOL implantation in which at least one haptic was placed in the ciliary sulcus were included in the study. All of these cases had open angles and heavy pigmentation on the trabecular meshwork in gonioscopy. Patients who had any history of uveitis, pigment dispersion syndrome, or glaucoma prior to undergoing the cataract operations were excluded. The cornea, iris, lens, and angle status of the fellow eyes were all evaluated by slit-lamp examination and gonioscopy to rule out the diagnosis of primary pigmentary glaucoma. In patients whose gonioscopy showed peripheral anterior synechiae of more than 90 degrees, or who were followed up for fewer than 6 months were also excluded. The main outcome measures were the onset of pigmentary glaucoma and the visual results of these cases following medical and surgical glaucoma treatment. External eye photography was performed to record the position of the IOL. Ultrasound biomicroscopy (UBM) was performed to establish the IOL position in the sulcus and to identify any possible contact between the IOL and the iris. Gonioscopy was performed to document both the status and the pigmentation of the angle. Visual field testing was performed to document visual field defects after the onset of glaucoma.

The patients’ records were reviewed to collect the following data: the initial IOPs (defined as IOP at referral) and final IOPs (defined as IOP at last visit); the initial visual acuity (VA) at referral and the final VA at the last visit; any visual field defects; the axial length; the presence or absence of an IOP elevation more than 21 mmHg during the early post-operative period; time of IOP elevation (defined as the time that elapsed between the initial cataract operation and the onset of chronic IOP elevation more than 21 mmHg); the follow-up period (defined as the time from the referral visit to the last visit); the type of IOL that was used; the presence or absence of vitreous incarceration around the IOL; the decentration of the IOL; the presence of chronic iridocyclitis; the method(s) of IOP control; and the visual outcome. Numerical variables are presented as the means and standard deviations (mean ± SD).

## Results

A total of 10 eyes of 10 patients were enrolled in this study. All of included eyes were referral cases from other clinics or hospitals. All of the included cases were Chinese. The mean age of the patients was 59.6 ± 10.7 years (range: 39–75 years). Six patients were men, and four patients were women.

**Figure 1:**
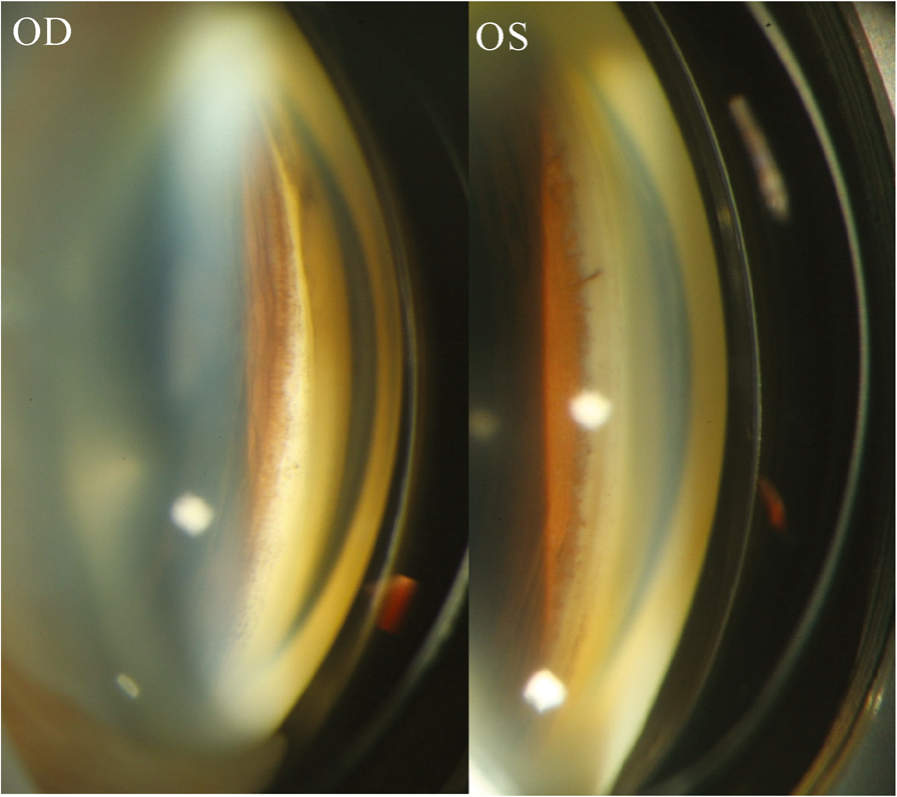
**Pigmentation of the angle structure after the implantation of a foldable intraocular lens (IOL) in the ciliary sulcus of one patient.** Compared with the angle of the fellow eye (OS), the angle in the eye in which the IOL had been implanted in the sulcus (OD) had much heavier pigmentation.

**Figure 2:**
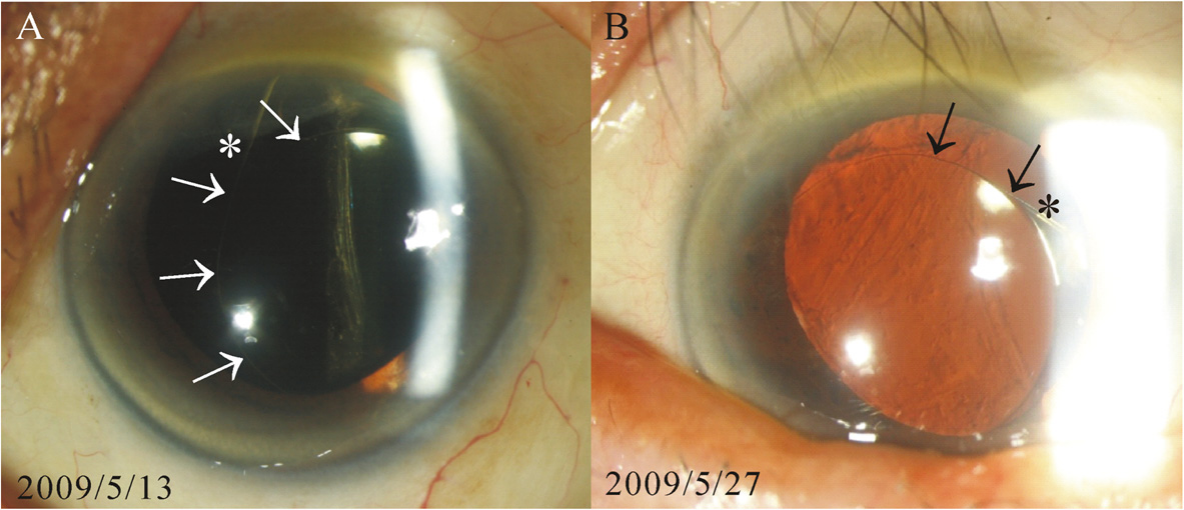
**Different intraocular lens (IOL) positions examined on different dates following placement in the sulcus.** The position of the IOL had shifted between the earlier and later examinations. The locations of both the margin of the IOL (arrows) and the IOL haptic (asterisk) had changed between the 2 examination dates (**A** and **B**). The shifting position of the IOL could have caused the dispersion of iris pigment and chronic inflammation of the eye.

**Figure 3:**
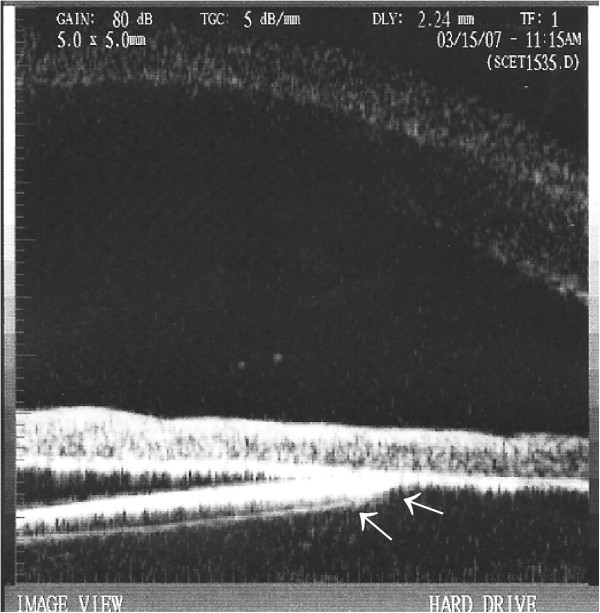
**Contact between the intraocular lens (IOL) and the iris demonstrated via ultrasound biomicroscopy (UBM).** UBM revealed that the optic of the IOL had direct contact with iris in this case (arrows), in which the IOL tilted because one haptic had been placed in the capsular bag and the other haptic had been placed in the sulcus.

Two different types of IOLs were used in the patients: 9(90%) one-piece foldable IOLs (Acrysof SA 60AT or SA30AT; Alcon, Fort Worth, Texas, USA), and 1 (10%) silicone IOL (CeeOn 911A, Pharmacia and Upjohn, Kalamazoo, Michigan, USA). At least one haptic of the IOL that was implanted in each eye was placed in the ciliary sulcus. The average time that elapsed between the implantation of the IOL and the onset of IOP elevation was 21.9 ± 17.0 months for all of the cases, and it ranged from 0.5 to 48 months. IOP elevation more than 21 mmHg within the 2 weeks after the initial cataract operation that constituted the early post-operative period was only observed in 2 cases (20%). One of the cases had persistent IOP elevation. The IOP of the other patient was soon put under control by anti-glaucoma medications, and he did not attend follow-up appointments until he experienced a second IOP elevation 4 months later. The other 8 eyes (80%) had delayed glaucoma onset, with the time between the initial cataract operation and the onset of glaucoma ranging between 4 and 48 months. The average period of time between the implantation of the IOL and the onset of glaucoma symptoms in these late-onset cases was 27 ± 15.3 months. Heavy pigmentation was observed in the angles of all of the diseased eyes (Figure 1). Pigment deposit was also observed on the corneal endothelium and the IOLs. Vitreous incarceration around the implanted IOL was found during the examinations of 3 of the 10 eyes (30%), and lens subluxation was noted in 2 eyes (20%). IOL was noted to have different positions at different times during the follow-up period in 1 eye (10%) (Figure 2). Chronic recurrent iridocyclitis was observed in 6 of the eyes (60%) during the follow-up period. UBM showed iris-IOL chafing in all of the cases included in the study (Figure 3). The demographic and clinical characteristics of the patients are shown in Table 1.

**Table 1:** Demographic and clinical characteristics of 10 patients

Eye (R/L) (no.)	5/5
Sex (M/F)(no.)	6/4
Age (year)	
Average	59.6 ± 10.7
Range	39-75
Type of IOL(no.)	
One-piece foldable IOL	9
Silicon IOL	1
IOL subluxation (Yes/No)(no.)	2/8
Chronic iridocyclitis (Yes/No)(no.)	6/4
Time of IOP elevation (months)(no.)*	
< 2 weeks	2
2 weeks-12 months	2
12-24 months	2
>24 months	4
Vitreous incarceration (Yes/No)(no.)	3/7

*F* female, *IOP* intraocular pressure, *IOL* intraocular lens, *M* male, *m* months, *no.* number, *R* right eye, *L* left eye.

*following cataract surgery in which an IOL was implanted in the ciliary sulcus.

All of the patients had IOPs that were greater than 21 mmHg at the onset of glaucoma. The mean initial IOP was 35.1 ± 7.6 mmHg (range: 25-51 mmHg). The average range of IOP fluctuation during different visits in these patients was 12.1 ± 7.0 mmHg, with a range from 4 to 21 mmHg. Four eyes (40%) were controlled with medication(s) alone, and, due to large IOP fluctuations (13 ± 5.2 mmHg) and poor control of the IOPs of the 6 remaining eyes (60%), these patients were treated with additional surgical procedures, which included replacing the foldable IOL with a polymethylmethacrylate (PMMA) IOL (3 cases), anterior vitrectomies, or exchange of IOL combined with trabeculectomies that were enhanced with the use of mitomycin C (3 cases). After the various treatments, 2 eyes (20%) maintained their initial VAs, 3 eyes (30%) had improved vision, and 5 eyes (50%) had poorer VAs compared with their initial VAs. Only 3 eyes (30%) achieved final VAs better than 20/40. The mean follow-up period was 46.3 ± 21.3 months (range: 20–76 months). The treatment outcomes and the visual prognoses of the patients are shown in Table 2.

**Table 2:** Treatment outcomes and visual prognoses of patients with pigmentary glaucoma following the implantation of an intraocular lens in the ciliary sulcus

**Patient no./eye no.**	**Initial IOP (mmHg)**	**Final IOP (mmHg)**	**Initial VA**	**Final VA**	**Management**	**F/U period (months)**
1	33	12	FC	20/222	IOL exchange + anterior vitrectomy	29
2	39	8	20/400	FC	Medical treatment	41
3	32	19	20/32	20/32	Medical treatment	47
4	31	10	20/100	20/50	IOL exchange + anteriorvitrectomy	47
5	51	12	20/32	20/200	IOL exchange + anterior vitrectomy + trabeculectomy	72
6	34	13	20/22	20/63	IOL exchange	20
7	35	12	20/22	20/32	IOL exchange + trabeculectomy	72
8	45	14	20/400	20/100	IOL exchange + trabeculectomy	10
9	26	16	FC	FC	Medical treatment	49
10	25	16	20/63	20/285	Medical treatment	76

*IOP* intraocular pressure, *VA* visual acuity, *Initial IOP* IOP at referral, *Final IOP* IOP in the last visit, *Initial VA* VA at referral, *Final VA* VA in the last visit;

*FC* fingers count, *F/U* follow-up, *HM* detects hand motion, *IOL* intraocular lens, *m* month, *mmHg* millimeter of mercury, *no.* number.

The phakic status of the fellow eyes included 5 phakic eyes and 5 pseudophakic eyes. The gonioscopic finding of these fellow eyes all showed an open angle with light pigment. The average IOP of the fellow eyes was 13.0 ± 3.3 mmHg. None of these fellow eyes showed signs of glaucoma.

## Discussion

Our study has shown that secondary pigmentary glaucoma accompanying the implantation of a foldable IOL in the ciliary sulcus may present as an acute IOP elevation during the early postoperative period in a small percentage of patients (20%). Late-onset IOP elevation with glaucomatous optic nerve damage was observed in the majority of the cases (80%). Most previous articles about the development of secondary pigmentary glaucoma following the implantation of an IOL in the ciliary sulcus reported early onset of post-surgical IOP elevation [7,11,12]. Some cases may have had normal IOPs or may have presented with transient IOP elevations during the initial postoperative period and were soon controlled by anti-glaucoma medication. These cases could present as acute IOP elevation with chronic glaucomatous optic nerve damage at a later date. At later stages in our study, large IOP fluctuations even with medical control were noted in 60% of the cases. This observation may be related to changes in the position of the IOL within the sulcus, and surgical interventions are necessary to control the IOPs in the patients. Cases with chronic IOP elevation that is not responsive to treatment with anti-glaucoma medications may require the replacement of the IOL, and/or a trabeculectomy with mitomycin C to control the IOP. Anterior vitrectomy might be needed to release the vitreous incarceration of the IOL during the exchange of IOL. Despite the use of various treatments, the visual prognoses for these patients could be poor in an advanced stage of glaucoma. Therefore, the implantation of a foldable IOL in the ciliary sulcus may pose a risk of developing late-onset pigmentary glaucoma, and performing this procedure after the rupture of the posterior capsule during a cataract surgery should be reconsidered.

Two previous articles [13,14] that support the use of a single-piece acrylic IOL in the sulcus have been published. However, other studies [7,9,15,16] have reported that this kind of IOL may induce surgical complications ranging from iris chafing and uveitis-glaucoma-hyphema (UGH) syndrome to vitreous hemorrhage. Uy and Chan [8] found evidence of associations between the implantation of sulcus-fixated, single-piece hydrophobic acrylic intraocular lenses and pigment release and secondary glaucoma in 20 eyes. They found that 7 eyes [35%] developed pigment release and 3 eyes (15%) developed secondary glaucoma. Chang et al. [9] report the largest case series (30 cases) published to date of patients with chronic complications resulting from sulcal placement of single-piece acrylic IOLs. In addition to pigment dispersion syndrome, they found evidence that complications such as recurrent iridocyclitis with secondary IOP elevation, intraocular hemorrhage, cystoid macular edema (CME), and lens decentration with symptomatic edge glare can occur [9]. However, as the average follow-up duration in Uy and Chan’s study [8] was only 17.2 ± 9.4 months, the incidence could be higher if those patients had been followed up over a longer period.

The vision of the patients in our study seems to be much worse than that of patients in previous studies. Only 3 eyes (30%) achieved final VAs of better than 20/40. Chang et al. [9] reported that the mean corrected distance visual acuities of the patients in their study improved postoperatively, resulting in the majority of eyes eventually attaining an acuity of 20/20. Uy and Chan [8] described postoperative best-corrected VAs of 20/40 or better in all of the eyes with pigment release and secondary glaucoma. In light of these findings from other studies, we propose some hypotheses regarding the poor vision of our patients. First, all of our patients were referral cases, and 6 of the eyes (60%) presented with visual acuities that were poorer than 20/40 at the time of referral. The high IOP at referral, corneal edema, lens subluxation and advanced glaucomatous optic nerve damage could be the causes of poor VA at referral. The IOPs of these patients may have fluctuated before they became symptomatic with uncontrolled IOP elevation, and this in turn could have led to advanced glaucomatous optic neuropathy prior to referral. Two eyes had lens subluxation and 6 eyes had chronic iridocyclitis. Finally, fluctuations of the IOPs in these patients were frequently noted despite medical and surgical interventions. All of these factors can lead to poor visual prognoses of our patients.

IOP elevation in the patients with sulcal foldable IOL implantation may be resulted from multiple mechanisms, including post-operative inflammation, chronic iridocyclitis, vitreous incarceration and pigment dispersion. Early IOP elevation might mainly result from the inflammation associated with the improper management of surgical complications such as vitreous incarceration. At the later stage, IOP elevation in the patients with sulcal foldable IOL implantation may be resulted from chronic iridocyclitis and pigment dispersion. Iris chafing by the IOL can arise because of the closer contact between the IOL and the iris that results from the sulcal placement of the IOL [2-5,7-12]. In addition, the flexible haptics of a foldable IOL facilitate the rubbing-induced detachment of iris pigments. Finally, the IOL can still be moving around freely as a result of the foldable IOL having a relatively smaller diameter than that of the sulcus (Figure 2). This could subsequently induce a “pigment storm” resulting in pigment accumulation in the trabecular meshwork that might further compromise the trabecular outflow and give rise to glaucoma. The most common histopathologic finding among the patients in the study by Chang et al. [9] was that of pigment granules on the anterior surface of the IOL, with the greatest accumulation occurring on the haptic of the IOL as well as the peripheral optic and the haptic–optic junction. These findings are consistent with the notion of posterior iris chafing caused by the optic and the relatively thick flexible haptics, all of which have squared edges and unpolished side walls. In one study of an experimental pigmentary glaucoma [17] model, researchers demonstrated an acute and substantial lowering of the outflow facility of the trabecular meshwork after pigment particles had been infused into the anterior chamber. During an early stage of the disease, pigment can be phagocytosed by trabecular meshwork cells or migrated away via trabecular endothelial cells with the outflow facility returning to normal [17]. However, in advanced pigmentary glaucoma, the trabecular tissue becomes overwhelmed and there is a loss of endothelial cell migration accompanied by collapse of the trabecular beams and subsequent sclerosis of the tissue [18,19]. This finding is compatible with the clinical course of the late-onset of IOP elevation that was observed in our study.

To avoid the complication of secondary pigmentary glaucoma following cataract surgery with vitreous loss, management of the herniated vitreous body using a vitrectomy and choosing and implanting IOL properly are very important. In these cases, the implantation of either a PMMA posterior chamber IOL in sulcus or an anterior chamber IOL can be performed following the vitrectomy. Fixating 3-piece acrylic IOL with suture or capturing the optic within a well-centered capsulorhexisis is another option [9].

As far as we are aware, the present study represents the largest case series to examine pigmentary glaucoma following the implantation of foldable IOLs in the ciliary sulci of an Asian study population. The average follow-up of the patients is over 3 years. However, the study is still limited by its retrospective nature, variable follow-up periods, and small number of cases. In addition, no control group was available. Thus, in view of the aforementioned limitations, cause and effect could not be established in this study. Improper management in cases in which there was vitreous loss could also contribute the pigment dispersion in some of our cases. More data are needed to explore both the incidence of secondary pigmentary glaucoma following the implantation of a foldable IOL in the sulcus and the causal relationship between such a procedure and its complications.

## Conclusion

This study has shown that secondary pigmentary glaucoma following the implantation of a foldable IOL in the ciliary sulcus may present as an acute IOP elevation during the early postoperative period. Or more commonly, it can present as late-onset chronic IOP elevation accompanied by large IOP fluctuations and glaucomatous optic nerve damage during a long-term follow-up period. Surgical interventions are usually needed in these cases. Late-onset of glaucoma symptoms and IOP fluctuation may result in poor visual prognosis despite medical or surgical management. Implantation of a foldable IOL in the ciliary sulcus is therefore not recommended. Because late-onset pressure rise may occur in these patients, long-term follow up for these patients is necessary if such a procedure is to be performed.

## Informed consent

The study was performed with the guidelines required by Chang Gung Medical Foundation Institution Review Board.

## Abbreviations

IOL: Intraocular lenses; IOP: Intraocular pressure; UBM: Ultrasound biomicroscopy; VA: Visual acuity; PMMA: Polymethylmethacrylate; F: Female; M: Male; M: Months; No.: Number; OD: Right eye; OS: Left eye; FC: Finger count; HM: Detect hand motion; F/U: Follow up; mmHg: Millimeter of mercury.

## Competing interests

The authors declare that they have no competing interests.

## Authors’ contribution

SHLC made substantial intellectual contributions to the conception and design, acquisition of data, and analysis and interpretation of data and revising it critically for important intellectual content and given final approval of the version to be published. WCW was involved in drafting the manuscript, analyzing and interpreting the data and revising it critically for important intellectual content. SCW was involved in revising it critically for important intellectual content and giving final approval of the version to be published. All authors read and approved the final manuscript.

## Pre-publication history

The pre-publication history for this paper can be accessed here:

http://www.biomedcentral.com/1471-2415/13/22/prepub
